# Utilization of Treg Cells in Solid Organ Transplantation

**DOI:** 10.3389/fimmu.2022.746889

**Published:** 2022-02-04

**Authors:** Tanya Juneja, Maria Kazmi, Michael Mellace, Reza F. Saidi

**Affiliations:** Department of Surgery, State University of New York (SUNY) Upstate Medical University, Syracuse, NY, United States

**Keywords:** T-regs, immunosuppression, transplant, kidney, rejection, solid, organ

## Abstract

Organ transplants have been a life-saving form of treatment for many patients who are facing end stage organ failure due to conditions such as diabetes, hypertension, various congenital diseases, idiopathic diseases, traumas, and other end-organ failure. While organ transplants have been monumental in treatment for these conditions, the ten year survival and long-term outcome for these patients is poor. After receiving the transplant, patients receive a multi-drug regimen of immunosuppressants. These drugs include cyclosporine, mTOR inhibitors, corticosteroids, and antibodies. Polyclonal antibodies, which inhibit the recipient’s B lymphocytes, and antibodies targeting host cytokine inhibitors which prevent activation of B cells by T cells. Use of these drugs suppresses the immune system and increases the risk of opportunistic pathogen infections, tumors, and further damage to the transplanted organs and vasculature. Many regulatory mechanisms are present in organs to prevent the development of autoimmune disease, and Tregs are central to these mechanisms. Tregs secrete suppressive cytokines such as IL-10, TGF-B, and IL-35 to suppress T cells. Additionally, Tregs can bind to target cells to induce cell cycle arrest and apoptosis and can inhibit induction of IL-2 mRNA in target T cells. Tregs also interact with CTLA-4 and CD80/CD86 on antigen presenting cells (APCs) to prevent their binding to CD28 present on T cells. Due to their various immunosuppressive capabilities, Tregs are being examined as a possible treatment for patients that receive organ transplants to minimize rejection and prevent the negative outcomes. Several studies in which participants were given Tregs after undergoing organ transplantations were reviewed to determine the efficacy and safety of using Tregs in solid organ transplantation to prevent adverse outcomes.

## Introduction

Solid organ transplantation is the treatment of choice for many patients who face end-stage organ dysfunction (1). Renal transplantation, for example, is the ideal option for patients facing kidney failure. Although there are clear short term improvements seen in patients post-transplant, the long-term survival of these patients is suboptimal due to morbidity and toxicities associated with immunosuppressive regimens and chronic rejection (1). After the patients have undergone transplant surgery, they are required to take various immunosuppressive drugs, which are necessary to decrease immune-related attacks of the transplanted organ. However, these drugs may pose other risks for transplant recipients. Examples of immunosuppressive therapies include calcineurin inhibitors such as tacrolimus and cyclosporine, antiproliferative agents such as mycophenolate mofetil, and mTOR inhibitors such as sirolimus. Immunosuppressive treatments work to prevent rejection and graft vs host disease by reducing the inflammatory response to prevent immune attack of the donated organ, which is seen as foreign. In graft vs host disease, there can be both an alloreactive response, when donor cells respond to recipient cells, and an autoreactive response, when donor immune cells respond against donor cells such as platelets or red blood cells (2). Immunosuppressive therapies prevent this but are often very expensive and have numerous side effects, such as greater susceptibility to severe infections and inability to respond adequately to pathogens (1). Due to the combination of immune reactions to organs and drug toxicities, the ten year organ survival rates of organ transplantation have not been very promising.

Transplant recipients receiving immunosuppressive treatments have been shown to be affected by non-Hodgkin lymphoma, lung, kidney, and liver cancers more frequently than the general population (3). They are also more susceptible to oncogenic viruses such as Epstein-Barr virus and hepatitis B (2–4). For example, in the first year after a heart or lung transplant, infections are the main cause of death, and remain a major cause of death and rejection in following years (4). As a result, there are many lifestyle changes that transplant recipients must make to protect themselves, although the risk of rejection persists even with immunosuppressive treatments. Despite how integral organ transplants are, there is a large shortage nationwide. Over 112,000 people are on a national waiting list for organs, according to data from March 2020, and approximately 20 people die each day awaiting organ transplants (5). Additionally, these immunosuppressive drugs can cause a slew of other health issues such as diabetes, nephrotoxicity, hyperlipidemia, hypertension, cardiovascular diseases, and obesity (3). Evidently, these complications may lead to withdrawal of the immunosuppression leading to graft rejection and loss. Transplants offer the best chance of survival for those facing end organ damage, therefore, it is imperative to prevent the adverse effects of immunosuppressive therapies and loss of transplanted organs. The use of regulatory T cells (Tregs) in organ transplantation is being explored as an option to reduce side effects and rejection in place of or in addition to immunosuppressive treatments. Tregs may provide a new mechanism to improve mortality, graft rejection, and infection rate in these patients.

Tregs are a key component of the immune system: a complex biological system whose job is to eliminate responses to allergens and pathogens, as well as recruit and work alongside many other immune cells. However, as important as it is to eliminate pathogens, another essential component of the immune system is the ability to discriminate between foreign pathogens and the body’s own cells. T-cells and B cells are components of the immune system and are heavily involved in preventing the cascade of adverse events that occur as a side effect of organ transplantation (2).

Tregs come from a class of cells called CD4^+^ T cells. CD4^+^ T cells are divided into T regulatory cells (Tregs) and T helper cells (Th) cells (2). Th cells control the adaptive immunity by activating and interacting with other effector cells such as CD8+ cytotoxic T cells, B cells, and macrophages. One of the major responsibilities of T regs is to suppress particularly harmful or dangerous activities of the Th 1 cells. Tregs are from the same lineage as naive CD4+ cells and express various biomarkers such as FOXP3, CD25 (6).

Tregs produce cytokines such as TGF-B and IL-35 (1), which suppress other immune cells and downregulate other cytokines such as TH1 cytokines, MHC class II antigens, and stimulatory effects on other cells (7). Expression of the nuclear transcription factor Forkhead box P3 (Fox P3) is the defining feature that determines natural Treg development and function and is crucial for maintaining and suppressing the immune system (3). The FoxP3 Treg cells migrate to sites of inflammation and can suppress various effector lymphocytes, specifically Th1, Th2, Th17, and follicular Th (Tfh) cells (1). There are two categories of CD4+ Tregs: natural Tregs (nTregs) and induced Tregs (iTregs). Foxp3+ Tregs are unique in the sense that they can be induced in the periphery from non-Foxp3+ T cell precursors along with other cytokines including TGF-B and IL-10. Additionally, according to a paper conducted by the Journal of the American Society of Nephrology, the memory Tregs of tolerant recipients exhibited increased Foxp3 TSDR demethylation, expressed higher levels of CD30 and glucocorticoid-induced TNF-related receptor, and harbored greater suppressive properties than memory Tregs from patients with stable graft function (8). nTregs develop in the thymus during positive and negative selection. nTregs arise from progenitor cells in bone marrow, and develop in the thymus during a series of positive and negative selection. iTregs develop in the periphery from conventional CD4+ T cells following antigenic stimulation under tolerogenic conditions. Both types of Tregs can be used to downregulate the immune response and mediate peripheral tolerance and various inflammatory reactions. nTregs and iTregs require T cell receptor (TCR) engagement in order to function. They both have been used in various solid organ transplantation studies, as will be discussed in subsequent sections of this paper.

Furthermore, every T lymphocyte relies on the cytokine IL-2 for their survival and proliferation. However, when Tregs secrete their IL-2 receptor, CD25, Tregs deplete the stores of IL-2 for other lymphocytes, which curbs the survival of surrounding T lymphocytes (2). Additionally, Tregs express a surface molecule, cytotoxic T lymphocyte antigen 4 (CTLA-4), which is known to bind the costimulatory molecules CD80/86 with a higher affinity than its proinflammatory competitor CD28, which is expressed on conventional T effector cells. When CTLA-4 binds to CD80/86 and outcompetes CD28, it prevents T effector cell activation (2). Moreover, CD80/86 has been hypothesized to upregulate indoleamine 2,3- dioxygenase expression on dendritic cells. One of the dendritic cells’ main roles includes the catabolism of tryptophan, which suppresses immune responses through the generation of certain immunosuppressive molecules (2). The loss of cell surface expression of CD127 on CD4+CD25+ regulatory t-cells (Tregs) may be a useful marker to isolate Tregs. Combining both FOXP3 and CD127 could be an effective way to give better identification for patients with operational tolerance after liver transplantation (9). Evidently, because of their immunosuppressive capabilities, Tregs have hypothesized to play a role in prevention of auto-immune diseases such as asthma, allergies, and acute and chronic graft rejection of organs (2).

The goal of our paper is to give an overview of clinical studies, completed and ongoing, that test the effectiveness of Tregs in solid organ transplantation trials by examining their effects on chronic rejection, acute rejection, graft versus host disease, and other associated diseases. These studies also examined the safety of Tregs and a variety of infusion protocols, methods for extracting and expanding Tregs, and the ability to induce tolerance in patients without the use of immunosuppressive therapies. According to an article found in the Official Journal of the Transplantation Society, a lack of regulation by DN25hiCD4+ cells has also been suspected in several clinical conditions such as rheumatoid arthritis, multiple sclerosis, and graft versus-host disease (10). In addition to assessing the efficacy and effects of Tregs in solid organ transplantation, our paper also aims to preview future studies planned for Treg research and the direction that future studies are headed. The trials, summaries, and data on completed Tregs in solid organ transplantation are found in **Table 1**.

**Table 1:** Completed clinical studies using T-regs.

Study	Organ	Recipients	Dosage	Graft survival	Rejection	Phase		Study outcome
Applicability, Safety, and Biological Activity of Regulatory T Cell Therapy in Liver Transplantation (11)	Liver	9	A) Pre transplantation: 0.5-1 x 106 Tregs/kg (3 participants)	100%	0%	I		T-reg infusions are safe and tolerable for liver transplant patients.
			B) Three months after transplantation: 3-4.5 x 106 Tregs/kg (6 participants)					
A pilot study of operational tolerance with a regulatory T- cell based cell therapy in living donor liver transplantation (12)	Liver	10	T cells 3.39 ± 10^6^ /kg	100%	33.33%	I/IIA		Seven patients successfully discontinued the use of immunosuppressive agents. Three remaining participants developed mild rejection and resumed low-dose immunotherapy.
A Phase I Clinical Trial with Ex Vivo Expanded Recipient Regulatory T cells in Living Donor Kidney Transplants (13)	Kidney	9	A) 0.5 x 10^9^ Treg cells/recipient	100%	0%	I		Infusion is safe for use and studies should advance to phase II trials.
			B) 1 x 10^9^ Treg cells/recipient					
			C) 5 x 10^9^ Treg cells/recipient					
Polyclonal Treg Adoptive Therapy for Control of Subclinical Kidney Transplant Inflammation (TASKp trial) (14)	Kidney	3	320x10^6^ cells	100%	0%	I		Treg infusions were safe and were not associated with side effects or acute rejections. In addition, the Treg infusions showed a decrease in expression of inflammatory genes in 2 out of three of the patients
Feasibility, Long-term Safety and Immune Monitoring of Regulatory T Cell Therapy in Living Donor Kidney Transplant Recipient (15)	Kidney	12	A) 1×10^6^ kg/cells	100%	0%	I		Demonstrated the feasibility and safety of Treg use in solid organ transplantation and the need for future trials on investigating efficacy.
			B) 3×10^6^ kg/cells					
			C) 6×10^6^ kg/cells					
			D) 10×10^6^ kg/cells					
Regulatory T cells for minimising immune suppression in kidney transplantation: phase I/IIa clinical trial (16)	Kidney	11	nTreg	100%	0	I/IIa		Demonstrated the use of autologous nTregs was safe and feasible even in patients who had a kidney transplant and were immunosuppressed
			A) 0.5 x10^6^ fresh cells/kg					
			B) 1.0 x 10^6^ fresh cells/kg					
			C) 2.5-3.0 ×10^6^ fresh cells/kg					
A Clinical Trial With Adoptive Transfer of Ex Vivo-induced, Donor-specific Immune-regulatory Cells in Kidney Transplantation-A Second Report (17)	Kidney	16	total number of infused cells: 14.3 - 35.7 × 106 /kg BW	50%	50%		I/IIa	Autologous T reg infusion may induce alloimmune hyporesponsiveness, but complete cessation of immunosuppressive therapies could not be achieved and requires more study
			0.21 to 2.9 × 106 /kg BW of CD4+CD25+Foxp3+ cells					

**Figure 1:**
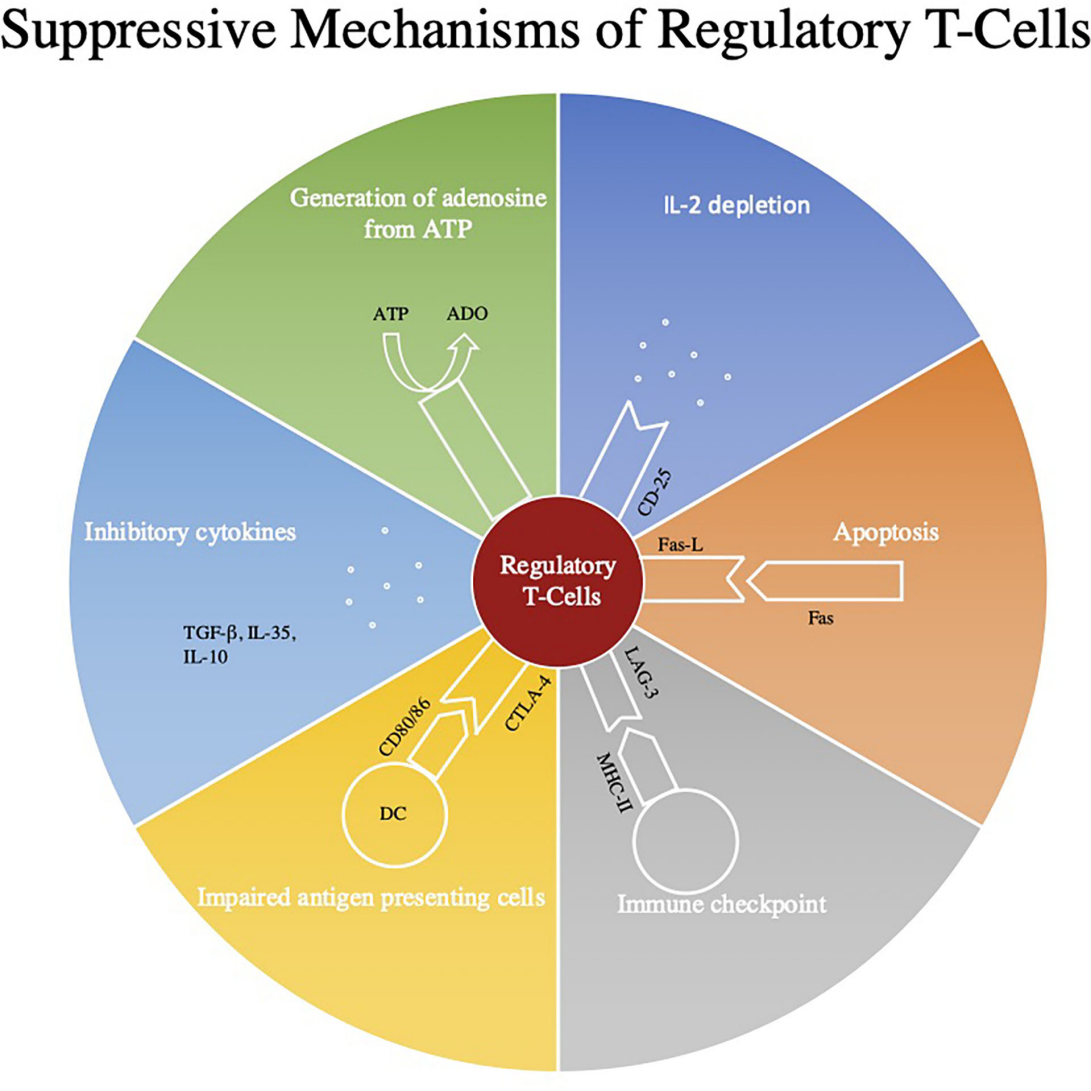
This figure represents some of the main regulatory mechanisms of Regulatory T-cells (Tregs). Firstly, through the use of CD39 and CD73 Treg’s convert ATP to adenosine which through A2aR signaling can inhibit the effects of CD8+ and CD4+ T-cells (18, 19). Next Tregs down regulate effector mechanisms by consuming cytokines such as IL-2. Tregs can also directly induce apoptosis by expressing Fas and Fas-L interaction and can modulate immune checkpoints by utilizing the LAG-3 and MHC II pathway. All three of these signaling pathways also induce apoptosis through the secretion of granzymes and perforins (20). Next Tregs can use cytotoxic T‐lymphocyte antigen‐4 (CTLA‐4) to impair modulate the maturation and function of antigen presenting cells (21). Lastly, they can secrete inhibitory cytokines such as TGF-β, IL-10, and IL-35.

T regs were used in various preparations displayed in **Figure 1**.

## Clinical Studies

Many studies have already been completed to examine the efficacy of the use of Tregs in organ transplantations, with respect to dosing, protocols, safety, and rejection rates. One example is a phase I pilot study of the applicability and biological activity of regulatory T Cell therapy in Liver Transplantation, conducted at King’s College in London. Nine patients were ultimately enrolled, with three enrolled pre-transplant and six enrolled six to twelve months post-transplant. The patients required liver transplants due to a variety of conditions, such as alcoholic cirrhosis, hemochromatosis, and hepatocellular carcinoma. Circulating Tregs were isolated from blood or leukapheresis, expanded under good manufacturing practices (GMP) conditions, and then administered intravenously to the participants in either 0.5-1 million Tregs/kg (3 participants) or 3-4.5 million Tregs/kg (6 participants) doses at least three months after liver transplantation (11). After infusion of the *polyclonal Tregs*, all participants were monitored using peripheral blood samples and continued with immunosuppressive protocols. Peripheral blood samples were taken nine times throughout the study, which was used for flow cytometric experiments or to isolate PBMCs. A Pleximmune test assay was used to quantify the number of recipient CD8^+^ CD45RO^+^ memory T cells expressing CD154 after 16 hours of culture with surrogate donor PMBCs. In the follow up period for all participants, there were no episodes of rejection and liver biopsies revealed normal histology, and the six participants that received 4.5 million Tregs/kg displayed donor specific hyporesponsiveness. According to the authors, due to small sample size, this cannot be considered a direct link to the Treg infusion, but may indicate a link that can be further studied. Ultimately, this trial was able to demonstrate the successful expansion of Tregs from patients undergoing immunosuppression and that Treg infusions are safe and tolerable for liver transplant patients (11). This would need to be verified using a larger sample size, as well as using wider criteria for selecting participants, such as those with previous transplants or autoimmune conditions, to determine if the use of Tregs is safe for all liver transplant patients. Additionally, with a larger number of participants, the use of Tregs could be analyzed in different conditions that led to patients receiving liver transplants, such as comparing the response in patients with a history of alcoholic cirrhosis to patients with a history of hepatocellular carcinoma prior to transplantation.

**Figure 2:**
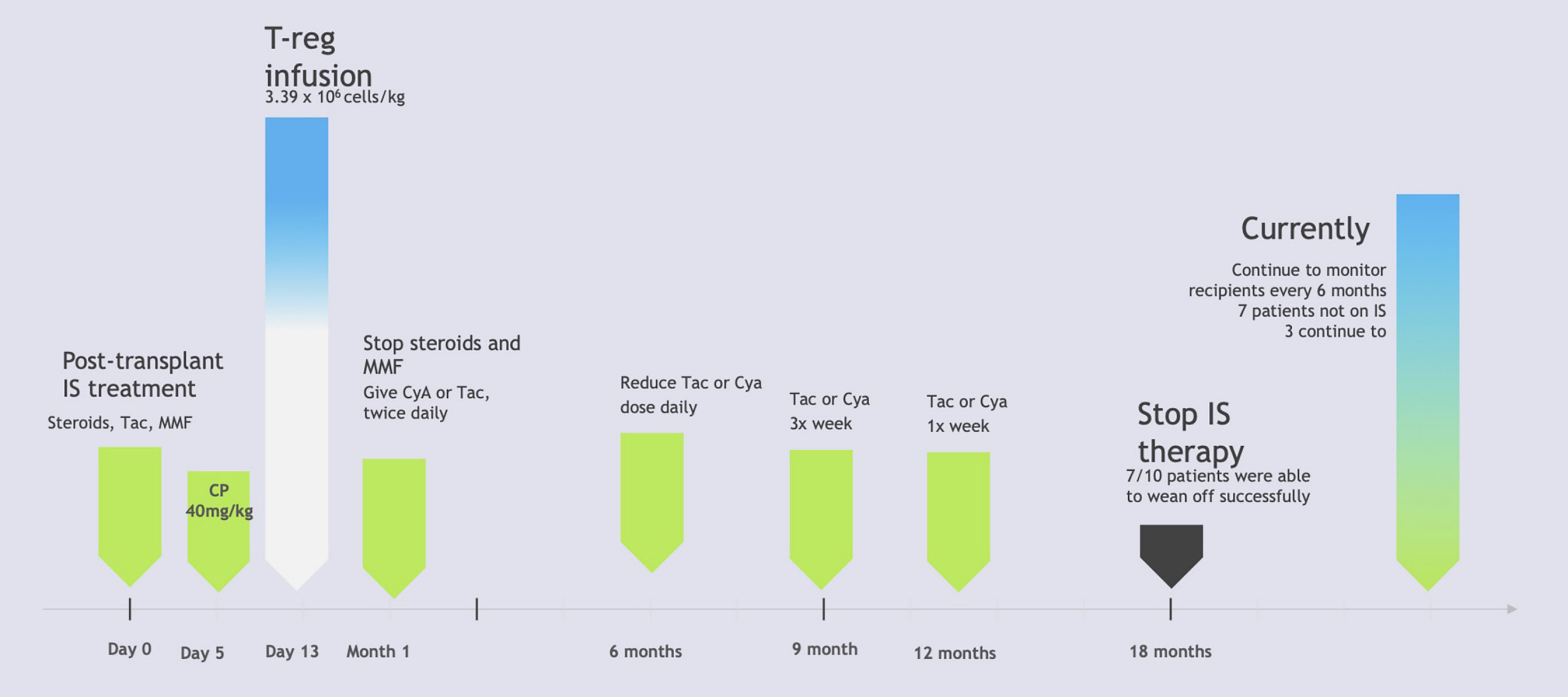
Liver Transplant Patients Immunosuppression Therapy Protocol. IS, Immunosuppression; Tac, tacrolimus; MMF, mycophenolate mofetil; CP, cyclophosphamide; CyA, cyclosporin A. Based on “A Pilot study of operational tolerance with a regulatory T-cell-based therapy in living donor liver transplantation” published in *Hepatology* by Todo et al. (9). This figure demonstrates the step by step protocol for patients who received regulatory T-cell therapy after undergoing liver transplants from live donors. Participants were monitored using liver biopsies as their immunosuppressive treatments were tapered. Ultimately 7 out of the 10 participants were able to successfully withdraw immunosuppressive therapy. The remaining 3 participants experienced signs of mild rejection and resumed immunosuppressive therapy protocols (9).

Another study was conducted in Japan, examining the operational tolerance with regulator T-cell based cell therapy in liver transplantations from living donors. This pilot study attempted to induce tolerance using regulatory T-cell based therapy in ten adult patients who received living donor liver transplants. T-cell enriched cell products were generated using a two week coculture of recipient lymphocytes with irradiated donor cells in the presence of anti-human CD80 and CD86 monoclonal antibodies. There was a three to six times increase in CD4^+^ Treg cells after culturing, along with an overall decrease in total lymphocytes. Following the liver transplantation, patients were given immunosuppressive therapy involving steroids and mycophenolate mofetil, which were discontinued within the first month post-op, along with tacrolimus. Rapamycin or cyclosporin A were used to replace tacrolimus in patients who experienced adverse reactions to other medications. Five days after transplantation, cyclophosphamide was also administered as part of the immunosuppressive therapy. The T-cell enriched cell products were given to patients in an infusion with normal saline thirteen days post-op. After being monitored for six months, the use of immunosuppressive therapy was tapered down with further reductions every three months, after eighteen months, and then immunosuppressive treatments ceased entirely. Liver biopsies were used to monitor graft function prior to dose reductions every three months, randomly if there were any signs of rejection, and every 6 months after treatment ended. Of the ten participants, seven were successfully weaned and able to discontinue the use of immunosuppressive agents, *achieving operational tolerance*, while the three remaining participants (who had autoimmune liver diseases) developed mild rejection during the weaning process and thus resumed low-dose immunotherapy. **Figure 2** illustrates the protocol used in this study. All the participants at the time of publishing were doing well and had normal graft function and histology (12). This study would be improved with a larger sample size with more variation in age of participants as well, as the patients’ ages ranged from only 39-63 years old. The study also only utilized patients who received transplants from relatives, which may have impacted the outcomes. This study was limited to only living-donor liver transplants and did not include any patients with deceased-donor liver transplants, which may reveal differences in patient responses to Tregs. An update of this study was recently published in 2017 which revealed that the seven patients who initially were weaned off immunosuppressive treatments continued to be tolerant almost five years later. These patients continued to maintain normal graft function at the time of their most recent evaluations, with immunohistological analysis revealing no progress of periportal lymphocyte infiltration or fibrosis (22).

In 2014, a phase 1 clinical trial of autologous polyclonal Treg Adoptive Cell Therapy (TRACT) in living donor kidney transplant recipients was conducted at Northwestern University. This study was nonrandomized with three tiers of cell dosing from 0.5, 1, and 5 × 10^9^ cells infused/recipient with three patients for each dosing tier (13). While this study chose diverse participants, the sample size of nine patients is very small. The mechanism for these thymus derived Treg cells is to control immune responsiveness to alloantigens and reduce graft versus host disease as has been shown in preclinical models. Pre-clinical models have shown that a high ratio of Tregs to CD4+ T cells such as 1:1 or 2:1 is necessary to achieve resistance to transplant rejection. In order to obtain this ratio, immunosuppressants were used to reduce the amount of CD4+ T cells and Tregs were later infused to reduce GVHD. Mycophenolate and tacrolimus were used for immunosuppression initially, with tacrolimus switched to sirolimus after one month. Tregs were then infused in patients at or after sixty days post-transplant. After Treg infusion, patients underwent immune monitoring of lymphocyte depletion and decrease in B cells, NK cells, and CD14^+^ monocytes, with recovery by day 90, while total CD4^+^ and CD8^+^ cells remained reduced. Monitoring also revealed a 5-20 fold increase in Treg percentages after Treg infusion in all participants, which was stable in most patients for the entire 1 year follow up period. The study focused on adults who were receiving living donor renal allografts and wouldn’t need hemodialysis in the first week after transplantation. This study found that there were no adverse side effects that could be attributed to Treg cell infusion. It is important to note that no opportunistic infections typically associated with generalized immunosuppression were noted, including nephropathy from polyoma virus and CMV. Patient and graft survival were also at a hundred percent two years later. This study thus concluded that an infusion of up to 5 × 10^9^ cells infused/recipient is safe for use and studies should be able to advance to phase II trials (13).

Additionally, in a study being conducted at University of California, San Francisco, three patients enrolled to receive a single infusion of 320x10^6^ cells ex vivo selected and expanded autologous polyclonal CD4^+^ CD127^lo/-^ CD25^+^ Tregs. The patients were given Basiliximab to induce immunosuppression and were given tacrolimus, mycophenolate mofetil, and prednisone. Two of the patients received transplants from living donors while one received a kidney from a deceased donor, and two of the patients had been on dialysis prior to surgery. The patients were infused at their six-month biopsy follow-up after renal transplantation. Follow up biopsies were conducted at two weeks and six months after infusion. The expanded Tregs were also marked with deuterium and tracked to determine stability of infused Tregs. This study, using the Banff scoring system for kidney allografts, showed that graft inflammation improved in the patients and there were no infusion reactions, infections, or acute rejections. Only one patient showed signs of subclinical acute rejection at the six month post-infusion biopsy but had reduced inflammation after his immunosuppressive medications were adjusted. Graft function also remained stable in all patients as they continued to be monitored (14). The study was able to achieve the isolation, expansion, and infusion of polyclonal Tregs successfully and safely in patients who received kidney transplants and underwent immunosuppressive therapy. This study was limited by a very small sample size but demonstrated the safety of polyclonal Treg infusion six-months after renal transplant, and could be repeated with a larger, more diverse sample size, since only three male patients were enrolled in this study.

In order to reduce the immunosuppression in organ transplantation, an international study called the One Study was conducted from 2012-2018 (23). This study involved seven separate investigative studies utilizing different mechanisms for reducing immunosuppression after organ transplants, conducted in eight hospitals worldwide. Each of these studies utilized cell-based medicinal products in live kidney transplant patients (23). Although kidney transplants have come a long way in the last fifty years, their efficacy is often complicated by adverse reactions against the transplanted organs. In order to combat the high immune reaction to the transplant organs, researchers are experimenting with various drugs to mitigate these adverse effects. The aim of the One Study is to decrease transplant organ rejection without the need for long-term pharmacological immunosuppression. It focuses on immune tolerance, manufacturing hematopoietic immunoregulatory cells, and tests cell therapy products in clinical trial living donor renal transplant rejections. In our review, we will focus on the trials in the One Study that utilize Tregs, due to their ability to produce immunosuppressive cytokines IL-10 and TGF-β (23).

A study that was a part of the ONE study took place in two different centers in the UK to try and explore the feasibility, safety, and potential efficacy of Treg therapy in kidney transplant patients. This study was designed as a prospective cohort phase 1 clinical trial. In this study twelve kidney transplant patients received a three plus three dose-escalation. Three patients at each dose received 1x10^6^, 3x10^6^, 6x10^6^ or 10x10^6^ cells per kg body weight at five days post transplantation, with a minimum of two weeks before escalation to the next highest dose (23). As a reference group, another nineteen patients received the standard immunosuppressive treatment. The primary outcomes measured were rejection rate, using the Banff criteria, and the survival of the transplant for sixty weeks post-surgery. In both groups the patient and transplant survival rates were 100%. All the patients received immunosuppressive therapy consisting of tacrolimus, mycophenolate mofetil, and prednisone, while the reference group additionally received induction with Basiliximab. The rejection-free survival rate was 100% in the Treg therapy patients, however, and only 78% in the standard immunosuppressive group at forty-eight months post-transplant. This study also found that the Treg-infused patients had a long lasting and dose-dependent increase in the amount of Tregs and in marginal zone B cell numbers. Additionally, 25% of the patients, who were CMV-negative, received CMV-positive transplants and none developed CMV infection after transplantation over the forty-eight month period. This study helped to further prove that Treg therapy is not only safe but can also have a lower rejection rate than standard immunosuppression therapy (15). This study could be improved with larger sample sizes and more diverse patients, as participants had strict exclusionary criteria in this study. Patients with a history of psychiatric conditions or patients with liver disease, for example, were not eligible for this study.

An additional trial was recently conducted in Germany as part of the One Study to assess if infusion of autologous nTregs in patients after kidney transplantation enables the tapering of immunosuppressive therapy. The study enrolled eleven patients who received living donor kidney transplants, who were compared to a reference group trial of nine patients. Autologous polyclonal nTregs were isolated and expanded from 40-50 mL peripheral blood samples with good purity and yield. The patients were given CD4^+^ CD25^+^ FoxP3^+^ nTreg products seven days after transplantation with one IV dose of either 0.5, 1.0, or 2.5-3.0 x10^6^ fresh cells, with three or four patients receiving each dose. Patients were given standard immunosuppressive regimens of steroids, mycophenolate mofetil and tacrolimus after transplantation as well. The patients were monitored to determine if nTregs allowed for the tapering of conventional immunosuppression from triple drug therapy to monotherapy within forty-eight weeks of transplantation.

Patients were studied for sixty weeks and then followed up at three years after transplantation for immune monitoring, assessment with biopsies to monitor for rejection, as well as clinical analysis of renal function. Eight out of eleven total patients were able to achieve stable monotherapy immunosuppression. Ten out of the eleven patients who received nTreg treatment were successfully weaned of immunosuppression to low dose tacrolimus monotherapy (trough blood levels <6 ng/mL) within forty-eight weeks, although tacrolimus monotherapy later failed in two patients. At the end of the sixty week study period, five of nine patients were on dual immunosuppression. At the three year follow-up, four of nine patients were on dual immunosuppression while the other five patients required triple drug immunosuppression. Ultimately, despite requiring additional immunosuppressive treatments, all of the eleven patients in this trial had good graft function at the three year follow up, as did the nine patients in the control group for comparison, although all required dual or triple drug immunosuppressive treatments. This study analyzed the safety, feasibility, and efficacy of nTreg treatment as well as monitored for drug interactions and adverse reactions. The use of nTregs in kidney transplant recipients was deemed safe and had no signs of immediate or long term over immunosuppression or adverse effects. Dose dependent affects were not seen, however, despite the use of three different doses of nTregs in the patients. This study was able to establish a procedure for isolation and expansion of autologous polyclonal nTregs with good purity and yield from a small blood sample of 40-50mL and demonstrate feasibility of this treatment (16). The study could be improved with larger sample size and more diverse patient population, as well as long term follow up of patients past the sixty month period used in this study.

Another study was conducting in Japan to attempt immune tolerance induction in kidney transplant recipients by infusing autologous donor-specific Tregs. This study recruited sixteen patients with end stage renal failure who had received kidney transplants from HLA mismatching living donors and split them into three groups for different preconditioning processes. In group A, nine patients underwent splenectomy on day 0 and received 25-30mg/kg/day of cyclophosphamide on day 5-7 for preconditioning. In group B, three patients were given 200 mg of rituximab was used in place of splenectomy, along with cyclophosphamide. The final four patients in group C were given 200mg of rituximab and 10mg/kg of rabbit antithymocyte globulin (rATG) from day 0 to 4 instead of cyclophosphamide. Preconditioning protocols varied in side effects, such as hair loss with the use of cyclophosphamide and post-op complications after splenectomy. All subjects had 5-8x10^9^ peripheral blood mononuclear cells (PBMCs) collected from the recipient and donor two days prior to transplantation. Recipient PBMCs were cocultured with 30 Gy-irradiated donor PMBCs with 12mg of antihuman CD80 and 12mg of antihuman CD86 monoclonal antibody. After seven days of coculture, viable cells were collected and cocultured again with 2x10^9^ 30 Gy-irradiated donor lymphocytes for seven more days. Finally, after fourteen days of coculture total, the viable cells were collected, washed, centrifuged, and suspended in 100mL of physiological saline. On day twelve post-transplant, the cultured cells were given to recipients intravenously, although the patients who could not be infused on day twelve due to transplantation complications were given the IV infusions later. The total number of infused cells ranged from 14.3 to 35.7 × 10^6^/kg BW and the amount of CD4^+^CD25^+^Foxp3^+^ cells were from 0.21 to 2.9 × 10^6/^kg BW. In the patients whose infusions were postponed due to postoperative complications, however, the cell number considerably decreased: 1.3 to 6.3 × 10^6^/kg BW in the total number of cells, 0.03 to 0.13 × 10^6^/kg BW in CD4^+^CD25^+^Foxp3^+^ cells (17).

All patients initially received immunosuppressive treatment 8 mg/kg of cyclosporine, 2000 mg/body mycophenolate mofetil and 500 mg/body of methylprednisolone daily. The doses of cyclosporine and mycophenolate mofetil were gradually reduced for the first three months. The dose of methylprednisone was also rapidly reduced to 20 mg/day on day 7, further tapered to 8 mg/day on day 14, and 4 mg/day on day 28, then gradually tapered off. The protocol aimed to reduce patient lymphocytes as little as possible. Patients were monitored using flow cytometry of peripheral blood samples and monitored serum creatinine for acute rejection. The patients in group A underwent early reduction of immunosuppressants with the goal of complete cessation at twelve months post-transplantation, however six out of nine participants experienced graft rejection within 1 year post-op. In group B, one out of three participants experienced rejection, and in group C one out of four participants experienced rejection. Complete cessation of immunosuppression was not achieved in any of the patients, however, and a low dose of immunosuppressive treatment was required. Ultimately, infusion of autologous ex vivo-expanded Treg was determined to be an option to possibly induce alloimmune hyporesponsiveness, but immune tolerance has not yet been achieved. This study revealed that further regimen modifications are required and may be studied in future trials until an ideal protocol is agreed upon (17). This study was limited by a small sample size and should be repeated with more patients to determine if the differing preconditioning protocols made a significant difference in rejection rates. Additionally, the study could be repeated with a more diverse patient population and further analyze if the specific pathology of kidney failure contributes to survival rates.

## Future/Ongoing Studies

While the aforementioned studies have shown some of the success of using Tregs as immunosuppressive therapy, many future trials may be needed to determine the best approach for using Tregs in different organs, with varying dosing, timing, etc. Currently there are many studies using Tregs as immunosuppressive therapy that are still ongoing and will be discussed further below. All of these studies will also be summarized and can be seen in **Table 2**.

**Table 2:** Future/ongoing clinical trials using T-regs.

Study	Organ	Recipients	Dosage	Phase	Goal	Status
Treatment of Children with Kidney Transplants by Injection of CD4+CD25+PoxP3+ T cells to Prevent Organ Rejection (24)	Kidney	30	2x10^8^ autologous T regs 1 month and 180 days after transplant	I	To assess the patient and graft survival of those receiving standard immunosuppressive therapy and those receiving Tregs.	Completed 2014. No results posted.
Liver Transplantation with Tregs at MGH (25)	Liver	9	2.5 x 10^6^ Tregs	I/II	Exploring cellular therapy to facilitate immunosuppression withdrawal in liver transplant recipients	Ongoing
A Pilot Study Using Autologous Regulatory T Cell Infusion Zortress (Everolimus) in Renal Transplant Recipients (26)	Kidney	12	Not stated	n/a	To determine the safety and effectiveness of collecting, expanding and infusing Tregs to kidney transplant patients undergoing immunosuppressive therapy.	Ongoing
The ONE study nTreg Trial (ONEnTreg13) (27)	Kidney	17	0.5 x 10^6^, 1 x 10^6^, and 2.5-3 x 10^6^ cells/kg body weight	I/II	Aims to explore the feasibility, safety, efficacy, of regulatory cell therapies using CD4+CD25+FOXP3+ nTregs in the context of living-donor renal transplantation.	Completed November 1, 2017. Awaiting results
Donor Alloantigen Reactive Tregs (darTregs) for Calcineurin Inhibitor (CNI) Reduction (ARTEMIS) (28)	Liver	14	400x10^6^ darTregs (range 300-500 x10^6^)	I/II	Assess incidences of adverse events associated with darTreg infusion, incidence of infection and malignancy. This study will also measure the number and proportion of individuals who are able to reduce calcineurin inhibitor dosing by 75%.	Completed 2019. Awaiting results
Treg Therapy in Subclinical Inflammation in Kidney Transplantation (29)	Kidney	33	Single infusion of 550 ± 450 x 10^6^ polyTregs	I/II	The goal of this study is to assess the efficacy of Tregs to reduce graft inflammation.	Ongoing
The ONE Study UK Treg Trial (ONETreg1) (30)	Kidney	15	1-10 million cells/kg	I/II	Explore the potential benefits of Treg therapy as an adjunct with immunosuppressive treatment in living donor transplants.	Completed March 3, 2017. Awaiting Results
Oxford Two Study (31)	Kidney	Minimum 34	Not stated	II	Achieve monotherapy with sirolimus and Tregs for kidney transplant patients in order to prove the protective effect of Tregs on kidney transplants.	Ongoing/Recruiting
Infusion of T-Regulatory Cells in Kidney Transplant recipients (The ONE Study) (32)	Kidney	5	N/A	I	Measure the safety and feasibility of administering Treg cells derived from a patient and administered alongside belatacept.	Completion date: March 2, 2016, Still Awaiting results

N/A, Not applicable.

A phase 1 study at Pirogov Russian National Research Medical University is aiming to assess the difference between patient and graft survival of those receiving standard immunosuppressive therapy and those receiving Tregs in pediatric kidney transplants patients. Participants were randomly assigned to receive either the full immunosuppressive therapy and autologous CD4^+^CD25^+^CD127 lowFoxP3^+^ T regulatory cells by subcutaneous injection (group 1) or immunosuppressive therapy alone (group 2). The full immunosuppressive therapy includes intravenous injection of alemtuzumab two to three weeks before transplant and on day zero after transplant. Starting on day zero, patients will then begin taking either tacrolimus or cyclosporine. Then on day three they shall begin mycophenolate mofetil. Prior to transplant, blood samples will be collected twice from patients with at least a one-week interval between collections, and the levels of T regs in the periphery will then be examined by flow cytometry in both groups (24).

The patients in group 1 will then undergo an injection of approximately 2 x 10^8^ autologous T regs expanded from previously frozen CD4^+^ cells after one month post-transplant and one hundred eighty days post-transplant. The measurement for the level of T regs in a patient’s blood will then be repeated in both groups after ninety and one hundred twenty days following transplantation. Using these measurements this study is looking to measure and compare the amount of Tregs in each group after treatment and transplantation. The patients will also be monitored for three years post-transplantation for graft rejection and patient survival. This study is currently listed as completed but the publication of results has not yet been posted (24).

There is also an ongoing clinical phase I/II trial at Mass General Hospital exploring cellular therapy to facilitate immunosuppression withdrawal in liver transplant recipients. The aim of this study is to measure the number and severity of adverse outcomes associated with donor alloantigen specific regulatory T cells. This study is also looking to address the number of operationally tolerant patients after fifty-two weeks of immunosuppression withdrawal (25).

In this study there are nine participants who will receive the target CD4^+^CD25^+^CD127 Treg cells (donor alloantigen-specific T regulatory cells), which is a single intravenous dose of 2.5 to 125 x 10^6^ cells post-transplant. Those who receive the minimum dose of 1 to < 2.5 x 10^6^ cells will be included in the intention to treat analysis. These cells will be obtained from the allograft recipient *via* leukapheresis seventy-two to one hundred twenty hours prior to infusion. Prior to receiving the Treg product patients will get a dose of 40 mg/kg cyclophosphamide and mesna. Patients will then be maintained on a Tacrolimus based immunosuppression regimen and will be eligible to switch to an everolimus based regimen between day thirty and week forty eight post-transplant, with a tapering off the tacrolimus once sufficient Everolimus levels are maintained (25).

Although a detailed method is not listed for this study, participants shall then be started on a trial of full immunosuppressive withdrawal. After participants are withdrawn from all immunosuppression, they will then undergo a research biopsy at fifty-two weeks to determine whether they meet the primary efficacy outcome of operational tolerance. Participants determined to be operationally tolerant will be followed for one hundred four weeks following drug discontinuation and will again have a research biopsy to confirm that they are operationally tolerant. In this study, in order to be considered operationally tolerant, three criteria must be met. First, a patient must be off immunosuppressive drugs for fifty-two weeks. Second, they must also have an alanine aminotransferase (ALT) and gamma-glutamyl transpeptidase (GGT) ≤ 50 U/L. Lastly, they must also have a liver biopsy result that is negative for rejection. This study began in March of 2019 and plans to end by March of 2026 (25).

Another study planned at the University of Kentucky is aiming to investigate/evaluate the safety and effectiveness of collecting, expanding and infusing autologous regulatory T cells to renal transplant recipients who are using Zortress (everolimus) as immunosuppressive therapy. The investigators are hoping to learn alternative ways of controlling the body’s immune response after renal transplantation and reduce adverse side effects of long-term immunosuppressive therapy. A detailed plan for this study has yet to be published but a brief overview of the methods has been explained. Investigators plan to enroll twelve individuals with end stage renal disease who are undergoing a solitary kidney transplant. Participants’ cells will be taken *via* apheresis and Tregs will then be expanded under special laboratory conditions for three weeks. Once a specified number of Tregs have been expanded they will be administered *via* a single intravenous infusion post-transplant. This study is primarily looking at toxicities twenty-four hours after infusion of Tregs, changes in kidney function two years post-transplant, rejection rates, and infectious complications throughout the study. This trial started in March 2019, and it plans to end by March 2021 (26).

As part of the ONE Study, at the University of Berlin, another phase I/II trial is aiming to explore feasibility, safety, and efficacy of regulatory cell therapies alongside other immunosuppressive treatments in renal transplant patients. It’s also aiming to see if it’s feasible to withdraw conventional immunosuppressive therapy within sixty weeks post-transplant after Treg infusion. This study has also not listed a detailed explanation of the methodology, but a brief overview was outlined. This study plans to recruit seventeen renal transplant patients to receive autologous CD4^+^CD25^+^FoxP3^+^ nTregs at escalating doses of 0.5 x 10^6^, 1 x 10^6^, and 2.5-3 x 10^6^ cells/kg body weight post-transplant in cohorts of three patients each. Participants will also be receiving prednisolone, mycophenolate mofetil, and tacrolimus. This study will plan to assess acute rejection, infections, pulmonary complications, organ failure, and problems associated with cell infusion. It also plans to measure outcomes of oversuppression of the immune system from Tregs by looking at incidences of neoplasia and infections from opportunistic organisms such as CMV, EBV, and polyoma virus. This study has not yet listed its plan or methods for immunosuppressive withdrawal. This study is listed as completed as of November of 2017 but there are no publications as of yet (27).

In a study from the National Institute of Allergy and Infectious Diseases (NIAID) a phase I/II trial is being conducted in order to see if it is safe for liver transplant recipients to receive a dose of donor alloantigen reactive Tregs (darTregs), and if it’s possible for these patients to then reduce or completely stop immunosuppressive medications normally taken after transplant. This study plans to recruit fourteen participants who have undergone liver transplantation to receive a dosage of 400x10^6^ darTregs (range 300-500 x10^6^) infused intravenously post-transplant (25). These patients will also receive a dose of 15 mg/kg of acetaminophen and 1-2mg/kg of diphenhydramine thirty to sixty minutes prior to the darTregs infusion.

Participants who fulfill the study criteria will then undergo withdrawal of immunosuppressive therapy. This includes subjects who have received at least 100 x 10^6^ darTregs no later than fourteen days post infusion and have ALT and either alkaline phosphatase or GGT within normal limits or less than or equal to 1.5 baseline post-transfusion. However, if participants do have elevated liver function, then they must have a liver biopsy within six to ten days of darTreg infusion that shows no acute rejection to still be eligible for withdrawal. Patients must also have entered the study on a calcineurin inhibitor or a calcineurin based inhibitor regimen with either prednisone or MMF. If the above criteria is met patients will undergo calcineurin inhibitor (CNI) withdrawal according to the CNI withdrawal algorithm. During the last two weeks of withdrawal patients will then receive a single unspecified dose of darTregs. If within two weeks of this infusion patients are eligible to continue past the seventy five percent withdrawal of baseline CNI, they will be offered a trial at complete withdrawal of immunosuppression.

This study will primarily measure the number of participants post-transplant and infusion with infections, with any malignancy, and who experience adverse effects deemed attributable to darTreg infusion. It also will look at the proportion of participants who were able to reduce their CNI dosing by seventy five percent and able to fully discontinue immunosuppressive drugs with stable liver function for greater than twelve weeks. This study has been listed as completed as of December 2019 but is now awaiting publication of the results (28).

In another phase I/II study from the National Institute of Allergy and Infectious Diseases (NIAID) researchers are looking to investigate if autologous polyclonal regulatory T cells (polyTregs) and darTregs can reduce inflammation in a transplanted kidney, and what the effects are on kidney transplant recipients. Another aim of this study is to determine the effects of taking everolimus alongside polyTregs post kidney transplant. Thirty patients will be split into one control group and one experimental group. The control group shall receive the standard maintenance CNI based immunosuppression with Tacrolimus, MMF (minimum dose of 1000mg per day), and Mychophenolic acid (minimum dose of 720mg per day). The experimental group will also receive the same immunosuppression regimen outlined above with the addition of a single infusion of 550 ± 450 x 10^6^ (at minimum 300 x 10^6^) of either darTregs or PolyTregs post-transfusion. Thirty to sixty minutes prior to transfusion patients will receive 25-50 mg of diphenhydramine and 650 mg of acetaminophen. Patients who have a target level of 4-11 μg/dl of tacrolimus will be eligible to have their dose reduced by fifty percent and start everolimus. Tacrolimus will then be discontinued four weeks after initiation of everolimus. This study will primarily observe the differences in the two groups by looking at the type/timing of rejection and infections if applicable. It will also look at the percent of inflammation in the two groups from biopsy and immunological profiles. Secondarily this study will also look at which subjects were and were not able to convert to MTOR inhibitor therapy and measure their timing/incidence of acute rejection. This study started in September of 2016 and is planned to be completed by June of 2022 (25).

Another study as part of the ONE STUDY is a phase I/II clinical trial whose aim is to assess cell therapy (autologous nTregs) as a treatment to prevent kidney transplant rejection. This study has recruited fifteen patients who are undergoing renal transplant from living donors. Although the method for nTreg extraction is not directly specified a brief overview is outlined. Each patient will have naturally occurring regulatory T cells isolated, grown in a laboratory, and re infused at a dose of 1-10 million cells/kg five days post renal transplant. Each patient will also be receiving prednisolone, MMF, and tacrolimus that will be slowly tapered after transplant. Patients will start with prednisolone 500mg IV on day zero, 125mg IV day zero to one, 20mg/day orally day two to fourteen, then 15mg/day orally weeks three to four, 10mg/day orally weeks five to eight, 5mg/day orally weeks nine to twelve, 2.5mg/day orally weeks thirteen to fourteen, and cessation of drug week fifteen. Next patients will receive MMF 500mg/day seven to two days before transplant, then a day before transplant will switch to 2000mg/day orally until fourteen days post-transplant, 100mg/day orally from weeks three to thirty six, 750mg/day orally from weeks forty one to forty four, 500mg/day weeks forty five to forty eight, and 250mg/day orally week forty nine to end with cessation of the drug. Next patients will receive tacrolimus 3-12ng/ml orally from four days pre transplant to fourteen days post-transplant, then 3-10ng/ml orally from weeks three to twelve, 3-10 ng/ml orally from weeks thirteen to thirty six, and 3-8 ng/ml orally from thirty seven weeks to end. This study will primarily measure the incidence of biopsy confirmed rejection within sixty weeks. This study states it began in April of 2014 and was completed in March of 2017 but is awaiting publication of results (30).

A separate study that is being planned as part of the “TWO study” plans to build upon the original ONE Trial by looking to see if nTregs can truly control rejection. In this trial they plan to isolate nTregs from thirty-four patients who are receiving either living or deceased renal transplants, grow them in a lab and reinfuse them into the patients. A detailed explanation of the methods of this study has yet to be outlined. The primary outcome to be measured is incidence of acute rejection episodes at twelve months post-transplantation. This study has yet to post a start or estimated completion date (31).

In another study conducted at Massachusetts General Hospital as part of the ONE Study, a phase I trial aimed to measure the safety and feasibility of administering Treg cells to renal transplant patients. In this study they recruited five participants who will first have blood collected from a donor and recipient. The cells were mixed together and also mixed with Belatacept. Then researchers plan to isolated regulatory T cells, and injected them back into the patient three days post-transplant. A detailed description of these methods that this study utilized have not yet been outlined. This study is primarily measured the safety and feasibility of Treg infusion with Belatacept in renal transplant patients within two weeks post-transplant. The specific measurements for how they will assess this safety and feasibility has not yet been outlined. This study has a listed start date of March 2014 and a completion date of March 2016 but results have not yet been posted (32).

## Discussion

One of the biggest challenges in transplantation currently is still allograft rejection. If we are able to overcome this, we will be able to improve outcomes for many patients. There has been a significant improvement in quality, results, and trials of organ transplantation with utilizing Tregs. Many of these differed in dosing, sample size, and methodology. Several studies as demonstrated in Table One such as the “Pilot study of operational tolerance with regulatory T-cell based therapy in living donor transplantation” and “Polyclonal Treg adoptive therapy for control of subclinical kidney transplant inflammation,” have shown the efficacy of Tregs in transplantation and how they can prevent adverse outcomes associated with transplant rejection (12, 14). For several trials, many participants who received the allogenic hematopoietic stem cell Treg infusion had decreased adverse effects associated with transplantation, such as decreasing graft vs host disease and improved graft outcome. As demonstrated in Table one, two of the three studies that used Tregs in patients undergoing liver transplantation showed a hundred percent graft survival rate (11, 12). While one study had no patients with rejection and the other had one third of patients with an episode of rejection (11, 12). Also demonstrated in Table one, there were five studies that used Tregs in kidney transplant patients with four studies showing a hundred percent graft survival and one study showing a fifty percent graft survival rate (13–16, 23). Four of the five studies also had no patients with an acute rejection episode with one study showing a fifty percent rejection rate 18, 19, 20, 21, 22). Overall, this shows that Tregs in transplantation have promising results.

In this paper we choose to focus on Tregs but there are also other clinical approaches currently under investigation to induce tolerance in solid organ transplantation. For example, instead of using Tregs to induce tolerance, studies are similarly using stem cells derived from bone marrow. In one such study, three patients underwent living donor living transplantation and then a week later underwent CD34+ stem cell transplantation (33). The results showed that two of the three patients were able to undergo early immunosuppressive withdrawal without subsequent graft rejection (33). The third patient however, had to rapidly resume immunosuppressive withdrawal after an attempt at discontinuation (33). None of the three patients were able to achieve chimerism, however these results demonstrated that donor stem cell infusion may help with early immunosuppressive withdrawal (33).

There are also case reports of donor stem cells being used to help induce tolerance in liver transplant patients but, instead of infusing them post-transplant, they are infused prior to transplant. For example, a case report was done that investigated the use of stem cell infusion to induce tolerance in HLA mismatched adult living donor liver transplants (34). In this study two patients were studied that underwent infusion of CD34+ stem cells prior to living donor liver transplantation (34). The results showed that one patient transiently observed chimerism while the other had no detection of chimerism. Both patients were ultimately successfully in achieving immunosuppressive withdrawal without a subsequent rejection episode and neither presented with GVHD (34). In a different case report, they attempted to sustain tolerance for solid organ allografts by first achieving full donor hematopoietic chimerism (35). They attempted this in two patients who first received hemopoietic stem-cell transplantation and then later received living donor liver transplant from the same HLA matched sibling donor (35). In both cases, patients were successfully weaned off immunosuppressants and have maintained full donor chimerism with normal graft function (35). In comparing these case reports to the two studies in **Table 1** where patients also underwent liver transplantation but with Tregs, the results are similar in that all patients had a hundred percent graft survival rate and decreased rejection rates (11, 12, 34, 35).

There are also other studies examining the use of bone marrow derived stem cells for tolerance in kidney transplant patients as well. For example, a study was conducted in sixteen kidney transplant patients using donor CD34+ selected cells alongside CD3+ T cells post-transplant (36). Of the sixteen patients, fifteen were able to achieve multilineage chimerism without GVHD. Eight of the patients with chimerism were able to be withdrawn from anti-rejection medications for one to three years without an acute rejection episode (36). In comparing this study to the first four studies in **Table 1** of patients who underwent kidney transplantation with Tregs, the results are similar in that there were no episodes of rejection (13–15, 23, 36). However, a comparable study was also done to analyze the long-term results of HLA-mismatched kidney transplantation without maintenance immunosuppression in ten subjects following kidney transplant alongside infusion of unprocessed donor bone marrow (37). All ten patients were able to achieve transient chimerism with seven patients able to successfully discontinue immunosuppression for four or more years (37). Ultimately four patients remained immunosuppressive free for four and a half to eleven and a half years while three required immunosuppressive treatment due to recurrence of original disease or rejection. The remaining three patients had allograft failure (37). Ultimately the results of using bone marrow derived stem cells to induce tolerance in organ transplantation has shown not only promising results but also like results to that of Tregs.

There are a number of future studies whose results and improvements need to be evaluated in order for further clinical use to occur. Further research studies with larger sample sizes and greater detail are needed to determine the true efficacy and utility of Tregs in solid organ transplants. The benefits and safety of Tregs may differ based on time of infusion, reason for transplant, and other conditions the patients may be affected by. While the studies reviewed demonstrate the great potential of using Tregs in solid organ transplant patients, there is a great deal more to learn before this is integrated into practice fully. When looking at the methods for the future/ongoing studies, it is apparent that there is currently not a standard procedure for using Tregs as immunosuppressive therapy. The timing, dosing, type of Tregs, immunosuppressive taper schedules, and whether to have concurrent use of immunosuppressive drugs alongside Tregs all differ amongst the different studies. As these studies are completed and results are published, it will not only be important to assess whether Tregs are effective, but also how each study’s method for use compares with each other to determine the best guidelines for use.

## Author Contributions

Each of the authors did an equal contribution to the work. TJ, MK, and MM all contributed equally to writing the introduction, organizing and creating the tables, as well as reviewing the future and ongoing studies. The authors also looked over each other’s’ work and brainstormed many ideas and shared articles to bring this review paper to completion. All authors contributed to the article and approved the submitted version.

## Conflict of Interest

The authors declare that the research was conducted in the absence of any commercial or financial relationships that could be construed as a potential conflict of interest.

## Publisher’s Note

All claims expressed in this article are solely those of the authors and do not necessarily represent those of their affiliated organizations, or those of the publisher, the editors and the reviewers. Any product that may be evaluated in this article, or claim that may be made by its manufacturer, is not guaranteed or endorsed by the publisher.
